# Tetanus Overlooked Due to the Involvement of Multiple Departments: A Case Report

**DOI:** 10.7759/cureus.48066

**Published:** 2023-10-31

**Authors:** Takanori Ohno, Masashi Kanazawa, Takaaki Nakano, Masaaki Takemoto, Toshitaka Ito

**Affiliations:** 1 Department of Emergency Medicine, Shin-Yurigaoka General Hospital, Kanagawa, JPN

**Keywords:** multiple departments, delayed diagnosis, developed countries, vaccination, tetanus

## Abstract

Tetanus is a fatal disease caused by a neurotoxin produced by the biotrophic anaerobic bacterium *Clostridium tetani*, which causes muscle hypertonia and autonomic neuropathy. The diagnosis is based on clinical findings and not the result of specific blood and imaging tests; hence, it is very difficult to diagnose at first sight, despite typical initial findings such as lockjaw, muscle spasms, and neck pain and stiffness.

This article discusses the case of a 79-year-old woman who first consulted her local doctor because of a lack of jaw opening. Seeing no improvement, she visited our hospital and was suspected of having tetanus after consulting with nine different departments over seven days from the initial visit. In developed countries, tetanus prevalence has declined due to immunization, leading to clinicians' lack of experience in diagnosing it. Furthermore, the increasing specialization in general hospitals poses a risk of missing a tetanus diagnosis when a patient consults multiple departments.

## Introduction

Tetanus is a fatal disease in which neurotoxins, produced by the disease-causing bacteria that invade the wound after trauma, spread hematogenously or lymphatically and derepress peripheral motor, cranial, and sympathetic nerves, causing muscle hypertonia and autonomic nervous disorders.

The incubation period between tetanus invasion and symptom onset can range from 24 hours to several months. The length of the incubation period is reflected by the distance the toxin travels from the wound site to the central nervous system and is related to the amount of toxins produced [1]. The period between initial symptom onset and the onset of convulsions is called the "onset time," and the shorter the incubation period and the onset time, the poorer the prognosis [1,2].

Therefore, it is advisable to start treatment for tetanus as early as possible, but it is often difficult to make a definitive diagnosis at the initial visit, with previous studies reporting a time from onset to diagnosis of 7.6 days [3].

The reasons for the delay in tetanus diagnosis include the low awareness of the usefulness of the spatula test (sensitivity 94%, specificity 100% [4]) and lack of experience among doctors in the developed world in diagnosing tetanus, as pointed out in previous reports [5].

In developed countries, vaccination against tetanus has been progressing, with the tetanus toxoid vaccine introduced in Japan in 1952 and the routine diphtheria-pertussis-tetanus combined vaccine, in 1968. Notably, of the 499 patients diagnosed with tetanus in 2018, 80% were aged 60 years or older [6]. Although the number of patients has declined, and tetanus has become a disease of the past, approximately 100 cases are reported each year in Japan. In a 2013 survey, the seroprevalence of the tetanus toxoid antibody was 5% in those aged >60 years (0.1 IU/mL) [7], and this age group also represents the unvaccinated population.

## Case presentation

**Figure 1:**
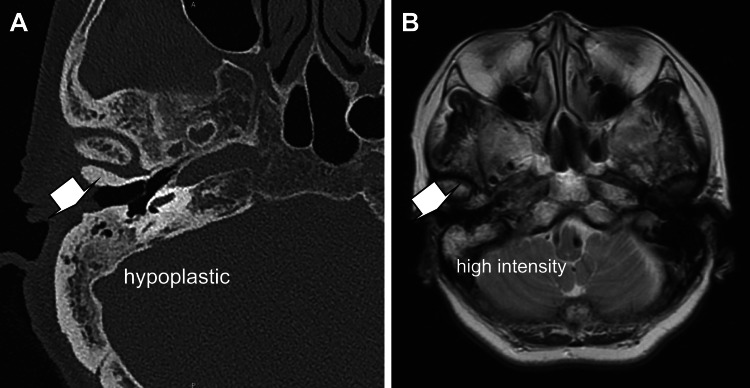
Computed tomography and magnetic resonance imaging of the head A. The right mastoid apicitis was hypoplastic, and chronic otitis media or mastoid apicitis was suspected. B. High-intensity findings in the right mastoid process on T2-weighted imaging suggest inflammation within the mastoid endoplasmic reticulum.

A 79-year-old woman with no previous medical history visited her primary care physician for symptoms that had appeared approximately two weeks prior to her visit, such as loss of voice and swallowing difficulty due to a lack of jaw opening, which meant that she could only consume soft foods. As there was no improvement after visiting the family doctor, the patient was referred to the department of otorhinolaryngology at our hospital (day -6), where she was diagnosed with a malfunction of the mouth and referred to the department of dentistry and oral surgery the following day. On the same day, she was also examined at the department of spinal cord surgery for neck discomfort, and a magnetic resonance imaging (MRI) scan of her neck showed no spinal disorder. The next day (day -5), the patient was referred by the otorhinolaryngologist to an oral surgeon and was deemed to have no problems with the temporomandibular joint, with an opening corresponding to 1.5 lateral fingers and a forced opening of 30 mm. The same day, on request for an internal medicine consultation, it was found that her weight had dropped from 48 kg to 43 kg over the previous two months. Additionally, she reported fatigue and imaging studies indicated a hypoabsorption zone in the left lobe of the thyroid gland. Subsequently, discharge was authorized. We decided to re-examine the thyroid hormones at a later date. Two days later (day -3), the patient visited the outpatient department of internal medicine, as she was unable to consume food orally. Dysarthria was suspected, and a computed tomography and MRI scan of the head were performed. The only finding was suspicion of right mastoiditis (Figure 1), therefore, the patient was sent home that day.

**Figure 2:**
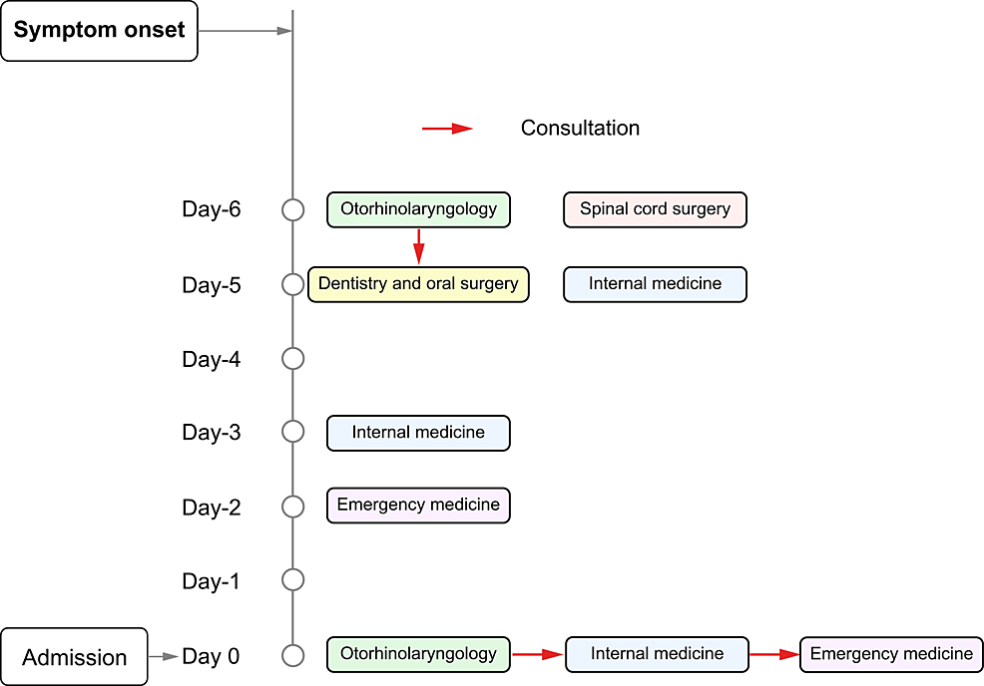
History of outpatient visits The first outpatient visit was six days before admission to the hospital. The red arrows indicate where one department had been consulted by another. A total of nine visits were made to five departments: otorhinolaryngology, spinal cord surgery, internal medicine, dentistry and oral surgery, and emergency medicine.

On the evening of the following day (day -2), the patient was admitted to the emergency department for a sense of dyspnea; however, since airway obstruction was not observed, she was sent home with a diagnosis of hyperventilation. Finally, at the return visit to the otolaryngologist (day 0), the possibility of tetanus was considered, and the patient again consulted the internal medicine department (Figure 2).

The consulting internist suspected tetanus, although there were no signs of trauma on physical examination, and the patient was referred back to the emergency department. Based on the clinical findings, tetanus was considered undeniable, and the patient was admitted for observation.

**Figure 3:**
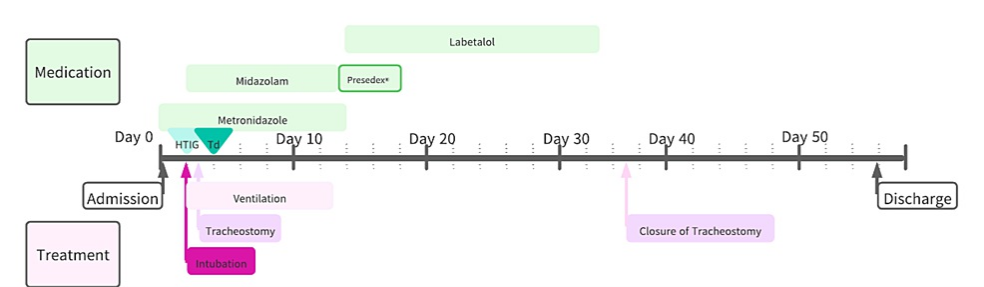
Timeline of tetanus treatment The generic name for Precedex® is dexmedetomidine hydrochloride. The date of admission is set as day 0, the medications used for treatment are listed in the top row, and the procedures performed are listed in the bottom row. HTIG: Human tetanus immunoglobulin was administered in 4500 units. Td: Tetanus toxoid was administered as a 0.5 mL intramuscular injection. Metronidazole was administered at 500 mg every 6 hours for 14 days. Labetalol 50 mg was taken internally three times a day from day 14 to day 35. 50 mg midazolam in 40 mL of saline was controlled 2 mL/H to 6mL/H from day 2 to day 13, then switched to dexmedetomidine hydrochloride on weaning from the ventilator and used until day 17.

After admission, the patient was started on metronidazole (500 mg, every 6 hours for 14 days) to treat tetanus. On the second day of admission, a single convulsive seizure occurred in the middle of the night, and intensive care management was started with intubation and ventilation. The seizure was judged to be due to tetanus, and 45000 units of human tetanus immunoglobulin were administered. Tetanus toxoid was administered on the third day of admission, and considering the possibility of long-term ventilation, tracheostomy was performed the day after ventilation was started (day 3 of admission). Midazolam followed by dexmedetomidine hydrochloride were used as sedatives during ventilation. The patient was on ventilation until day 13 of hospitalization, and although her circulation was stable on admission, by day 14, the increase in blood pressure due to stimulation was poorly controlled; hence, she was started on labetalol hydrochloride (150 mg/day), which she continued to until day 36. The complications that occurred during hospitalization included pneumonia due to the use of a respirator as well as heart failure and pulmonary embolism, none of which were disabling after discharge. The tracheostomy stoma was closed on day 38 of admission; the patient was discharged after 56 days when her general condition had stabilized, following the completion of rehabilitation treatment (Figure 3).

## Discussion

The isolation rate of tetanus is said to be about 30% because its causative agent, Clostridium tetani, is a biased anaerobe and cannot grow in the presence of oxygen [8]. According to the National Institute of Infectious Diseases in Japan, the organism could be isolated only in one of 157 cases of tetanus reported in 1999-2000 [9]. This means that the final diagnosis has to be based on clinical symptoms and the presence/absence of a trauma history, although, in about 30% of the patients, there is no clear history of trauma [1], which makes it difficult to definitively diagnose tetanus on the first visit. The Tokyo Metropolitan Centre for Health and Safety Research reported that the circumstances at the time of infection could be estimated in 34 patients, with the most common being gardening (including those described as gardening or fieldwork) in 12 (14.5%) and falls and bruises in nine (10.8%), while 49 patients had unknown sources of infection [8]. In the present patient, the infection was thought to have been triggered by gardening, but the long incubation period meant that no obvious trauma could be identified at the time of examination, which delayed the diagnosis.

The Tokyo Metropolitan Government, near our hospital, reported 83 cases of tetanus (5.9 cases per year) over a 14-year period from 2006 to 2019, which is about 5% of the total number of patients in Japan. In that study, only 17 patients (1.2 per year) were confirmed or estimated to have resided in a district that appeared to be close to our hospital [10]. Therefore, the fact that most of our doctors, who practice in urban areas, had no experience in treating patients with tetanus may have been a reason for the delayed diagnosis.

In a study conducted in South Korea, the diagnosis of tetanus could only be confirmed in emergency care in nine out of 17 patients (53%). The diseases in the differential diagnosis included temporomandibular joint disorder, temporomandibular joint and cervical dystonia, meningitis, cerebral infarction, spinal cord injury, and hypertensive encephalopathy [11]. Clinical symptoms range from head and neck symptoms, such as dysphagia, to spinal symptoms, such as gait disturbance and urinary and defecation disorders, and prior case reports have stated that this disease cannot be treated by only one specific department. In 2012, a report of 90 patients with tetanus in Japan stated that 22%, 19%, 18%, 9%, and 7% of the patients were treated by specialists in internal medicine, emergency medicine, otorhinolaryngology, surgery, and dental surgery, which did not necessarily mean that only one specific department was involved in the treatment of the patients at the time of the initial visit [12]. Although our patient had characteristic clinical conditions at the time of presentation, nine departments were involved over a period of seven days before the suspicion of tetanus was raised. The first otolaryngologist suspected mastoiditis based on the MRI findings. The spinal surgeons were concerned about spinal disease because of cervical discomfort, and the oral surgeons, who saw the patient the next day, underestimated the patient's oral dysphonia because he could open his mouth 30 mm by forced opening, and the policy was to refer her to a medical doctor. The internal medicine department also considered thyroid disease due to anorexia, weight loss, psychiatric disorders, and hyperventilation-like respiratory distress symptoms. The involvement of multiple departments in the treatment of tetanus seemed to have led to a tendency to focus only on the diseases that each department specialized in and eventually tetanus was overlooked by multiple departments.

If a patient presents with typical symptoms without a history of trauma, the likelihood of suspecting tetanus is reduced. Conversely, the number of doctors experienced in the treatment of tetanus is also low in developed countries due to the progress made in vaccination. Patients typically visit several relevant departments because their test results are unremarkable, but their symptoms do not improve. The combination of these factors is likely to be the cause of a vicious circle that delays the diagnosis of tetanus. To prevent missed tetanus diagnoses in developed countries, clinicians should receive regular tetanus education. Additionally, integrated healthcare teams involving multiple departments should collaborate to assess symptoms and make diagnoses, not just for tetanus but for various conditions.

## Conclusions

One of the problems related to tetanus diagnosis in developed countries is the lack of clinical experience with tetanus among doctors and the fragmentation of specialized diagnostic areas in acute general hospitals. These factors, including the involvement of multiple departments, can lead to the tetanus diagnosis being missed, even in patients with typical symptoms. However, considering that tetanus will continue to persist and patients may present to more than one department, collaboration between departments is essential for a timely and accurate diagnosis.
